# Stepwise Targeted Screening for Familial Hypercholesterolemia in a Resource-Limited Setting: A Scalable Model Linking Premature Coronary Artery Disease and Community Screening in Vietnam

**DOI:** 10.5334/gh.1557

**Published:** 2026-05-18

**Authors:** Thanh-Huong Truong, Doan-Loi Do, Minh-Phuong Vu, Mai-Ngoc Thi Nguyen, Hong-An Le, Thanh-Tung Le, Trung-Thanh Tran, Hong-Phu Vu, Dinh-Tuan Nguyen, Van-Dung Le, Ngoc-Thanh Kim

**Affiliations:** 1 Phenikaa University Hospital and Phenikaa School of Medicine and Pharmacy, Phenikaa University, Ha Noi, Vietnam; 2 Department of Cardiology, Hanoi Medical University, Ha Noi, Vietnam; 3 Vietnam National Heart Institute, Bach Mai Hospital, Ha Noi, Vietnam; 4 Department of Surgery, Hanoi Medical University, Ha Noi, Vietnam; 5 Department of Cardiology, Ha Tinh General Hospital, Ha Tinh, Vietnam

**Keywords:** Familial hypercholesterolemia, screening, premature coronary artery disease, community

## Abstract

**Background:**

Familial hypercholesterolemia (FH) is a common autosomal dominant disorder associated with a substantially increased risk of atherosclerotic cardiovascular disease. In low- and middle-income countries (LMICs), universal FH screening is often impractical, highlighting the need for targeted screening strategies embedded within existing health systems.

**Objectives:**

To evaluate the detection yield and real-world implementation feasibility of a stepwise targeted FH screening strategy in a resource-limited setting.

**Methods:**

This multi-component implementation study included retrospective and prospective hospital-based screening of patients with premature coronary artery disease (CAD), followed by a geographically targeted community-based screening program linked to a genetically confirmed FH index case in Vietnam. FH was assessed using the Dutch Lipid Clinic Network criteria, with LDL-C thresholds applied for community-level screening.

**Results:**

Among patients with premature CAD, phenotypic FH was identified in 2.5% and 8.3% of the retrospective and prospective cohorts, respectively. The higher detection rate in the prospective cohort may reflect more systematic phenotyping. In the community screening, 59.1% of eligible individuals participated, and 10.6% had LDL-C levels ≥ 4.9 mmol/L.

**Conclusions:**

A stepwise targeted FH screening strategy—initiated in clinical populations and extended via geographically targeted community screening—demonstrates feasibility and potential scalability in resource-limited settings and may provide a pragmatic framework for integration into national NCD programs in LMICs.

**Highlights:**

– Targeted screening strategies may enhance the detection of FH in resource-limited settings where universal screening is not feasible.
– Patients with premature coronary artery disease represent a high FH detection rate.
– Geographically targeted community-based screening linked to FH index cases enables identification of individuals with markedly elevated LDL-C levels.
– A stepwise approach combining hospital-based and community-based screening may provide a scalable model for FH detection in LMICs.

## Graphic abstract

**Figure:**
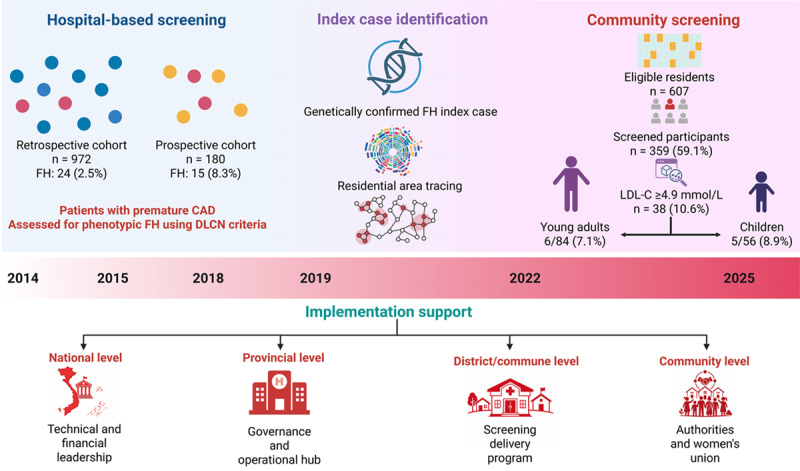

## Introduction

Familial hypercholesterolemia (FH) is an autosomal dominant disorder characterized by a lifelong elevation in low-density lipoprotein cholesterol (LDL-C) levels and a markedly increased risk of atherosclerosis. However, FH remains underdiagnosed worldwide; less than 10% of affected individuals are identified, leaving many exposed to preventable cardiovascular events (1). This diagnostic gap is particularly pronounced in low- and middle-income countries (LMICs), where limited healthcare resources, restricted access to lipid testing and genetic diagnostics, and low disease awareness impede systemic FH identification (2,3).

Within LMICs, screening strategies must balance diagnostic yield with feasibility. Universal screening is often difficult to implement and sustain. In contrast, opportunistic approaches that focus on high-risk clinical groups, particularly patients with premature coronary artery disease (CAD), offer a pragmatic entry point, given the substantially higher pretest probability of FH in this population. However, hospital-based detection alone is insufficient to address the broader burden of undiagnosed FH (4).

In Vietnam, multigenerational living and stable communities may promote geographic clustering of inherited disorders, even without formal genealogical records (5). This context creates an opportunity to extend targeted screening beyond clinical settings to identify communities linked to FH index cases. Therefore, we designed a stepwise targeted FH screening strategy that integrates hospital-based screening in patients with premature CAD with geographically targeted community screening linked to an FH index case. The present study evaluated the detection yield and real-world feasibility of this approach in a resource-limited setting.

## Methods

### Study design and settings

This multi-component, hybrid retrospective-prospective study combined (1) a retrospective hospital-based cohort, (2) a prospective hospital-based cohort, and (3) a community-based screening population in Vietnam.

Hospital-based screening (populations 1 and 2) was conducted at the Vietnam National Heart Institute (VNHI), Bach Mai Hospital, a national tertiary referral center. The retrospective and prospective cohorts were defined as separate study populations based on different enrollment periods and data collection approaches.

The third population comprised a community-based screening cohort implemented in Ha Tinh province through the Vietnam Familial Hypercholesterolemia (VINAFH) Program (6).

**Figure 1:**
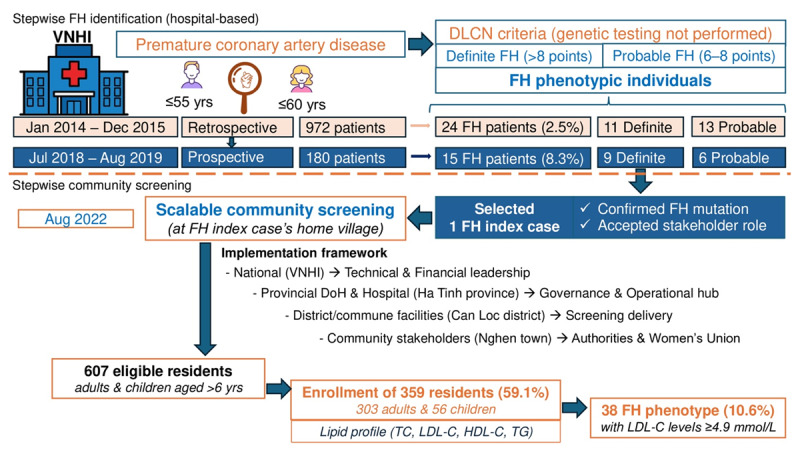
Timeline of stepwise identification pathway for familial hypercholesterolemia from hospital to community in a resource-limited setting. At the stepwise hospital-based FH identification, DLCN criteria (based on clinical data only) were applied. Only individuals with definite or probable FH proceeded to further evaluation. Subsequently, only patients with confirmed FH-causing mutations identified in the prospective cohort who agreed to act as stakeholders were selected for community-based FH screening. DLCN, Dutch Lipid Clinic Network; DoH, Department of Health; FH, familial hypercholesterolemia; HDL-C, high-density lipoprotein cholesterol; LDL-C, low-density lipoprotein cholesterol; TC, total cholesterol; TG, triglyceride; VNHI, Vietnam National Heart Institute.

The three populations were analytically integrated into a stepwise screening framework. Hospital-based cohorts were used to estimate FH detection yield among patients with premature CAD, whereas the community-based screening represented a linked extension after identifying a genetically confirmed index case. Analyses were primarily conducted within each component, with selected comparisons (prospective vs retrospective cohorts) to assess incremental yield. Community data were not pooled with hospital cohorts but interpreted as a complementary population-level extension. The overall screening strategy implemented is illustrated in Figure 1.

#### Step 1: Hospital-based screening in patients with premature CAD

##### Retrospective cohort

Medical records of adults (≥ 18 years) admitted to VNHI between January 2014 and December 2015 with a diagnosis of premature CAD (men ≤ 55 years, women ≤ 60 years) were reviewed. Patients with conditions that could confound lipid interpretation (liver or kidney disease, hypothyroidism, malignancy, pregnancy, and lactation) were excluded. All eligible patients were included without sampling.

##### Prospective cohort

Consecutive adults with premature CAD admitted to the VNHI between July 2018 and August 2019 were prospectively enrolled and underwent systematic FH assessment using standardized protocols.

#### Step 2: Community-based screening linked to an index case

##### Rationale and entry point

Community screening was implemented as an extension of hospital-based targeted screening after identification of a genetically confirmed FH index case in the prospective cohort. This case served as an entry point for geographically targeted screening in a long-term residential community considered at increased risk for FH. This approach is based on the known familial clustering of FH, an autosomal dominant disorder, in which affected relatives often share genetic risk and geographic proximity (semi-urban and rural settings). Geographic targeting around an index case serves a pragmatic extension of cascade screening, increasing the likelihood of identifying additional undiagnosed FH cases within the community.

##### Implementation framework and stakeholder engagement

The program was delivered through a pragmatic multilevel framework. The VNHI provides technical oversight, while provincial and local health systems coordinated field implementation, logistics, and recruitment in collaboration with community organizations.

##### Target population and screening procedures

A population-based, geographically defined sampling approach was applied. A registry of all residents in the index case’s long-term residential village was compiled with the local authorities. All eligible residents (aged > 6 years) were invited using a census-based approach to participate in a voluntary screening campaign in August 2022.

Participants underwent standardized clinical assessment and fasting lipid profile testing. Individuals with suspected FH phenotypes, defined by markedly elevated LDL-C levels (≥ 4.9 mmol/L) (7), were counseled and referred for further evaluation. Considering the resource-limited context and the scale of screening, LDL-C was used as a pragmatic first-line screening marker because of its feasibility, cost effectiveness, and established use for FH phenotype identification. Individuals exceeding the threshold were referred for comprehensive assessment (8).

##### Feasibility and participation

Among 607 eligible residents identified, 359 participated, corresponding to a participation rate of 59.1%.

### Data Collection and Measurements

Clinical data, family history, and lipid profiles were extracted retrospectively or prospectively using standardized case report forms. FH was assessed using the Dutch Lipid Clinic Network (DLCN) criteria. In community screening, an LDL-C threshold of ≥ 4.9 mmol/L was applied to identify individuals with the FH phenotype.

CAD was confirmed by coronary computed tomography angiography and/or invasive coronary angiography demonstrating obstructive disease (≥ 50% stenosis) in at least one major coronary artery. Patients with myocardial infarction were classified as having CAD regardless of stenosis severity, recognizing that plaque rupture or thrombosis may occur in non-obstructive lesions. Chronic CAD was defined as ≥ 50% stenosis. Premature CAD was defined as CAD occurring in men aged ≤ 55 years and women aged ≤ 60 years. Young adults and children were defined as individuals aged 18–40 and < 18 years, respectively. Multivessel disease was defined as ≥ 50% stenosis in two or more major coronary arteries (≥ 2.5 mm in diameter).

Phenotypic FH was assessed using the DLCN criteria, which integrate clinical features, family history, and LDL-C levels. The participants were classified as having unlikely (< 3 points), possible (3–5 points), probable (6–8 points), or definite FH (> 8 points). In community screening, LDL-C levels ≥ 4.9 mmol/L were used to estimate the burden of FH phenotypes and identify individuals requiring further evaluation (9).

### Statistical analysis

Data were summarized using descriptive statistics. Categorical variables are expressed as numbers and percentages. Continuous variables are presented as means ± standard deviation with 95% confidence intervals (95% CIs) or as medians and interquartile ranges (IQRs), as appropriate. Between-group comparisons were conducted using Student’s *t* test or Mann–Whitney U test for continuous variables and chi-square or Fisher’s exact tests for categorical variables, as appropriate. Odds ratios (ORs) and 95% CIs were calculated to assess the association between prospective screening and FH detection. All analyses were two-sided, with a p < 0.05 considered statistically significant, and performed using IBM SPSS Statistics for Windows, version 26.0 (IBM Corp., Armonk, NY, USA).

## Results

### FH detection in retrospective vs. prospective hospital-based screening

The FH detection yields and phenotypic classifications of patients with premature CAD who underwent retrospective and prospective hospital-based screening are summarized in Table 1. In the retrospective cohort, 24 of 972 patients were identified as having phenotypic FH (2.5%), whereas in the prospective cohort, 15 of 180 patients met criteria for phenotypic FH (8.3%). Prospective screening was associated with a significantly higher detection yield compared with retrospective screening (p < 0.001, chi-square test), corresponding to an OR of 3.59 (95% CI 1.84–6.99). The higher detection yield observed in the prospective cohort is partly attributable to more complete ascertainment and documentation of family history and physical signs (tendon xanthomas and arcus cornealis). Contrastingly, retrospective data were limited by incomplete documentation. The availability and distribution of selected DLCN components in both cohorts are presented in Supplementary Table S1.

**Table 1:** Detection yield of phenotypic familial hypercholesterolemia among patients with premature coronary artery disease in retrospective and prospective hospital-based cohorts.


CHARACTERISTICS	RETROSPECTIVE SCREENING (n = 972)	PROSPECTIVE SCREENING (n = 180)	P-VALUE

***Phenotypic FH, n (%)***	24 (2.5)	15 (8.3)	<0.001

*Definite FH (DLCN >8), n (%)*	11 (1.1)	9 (5)	<0.001

*Probable FH (DLCN 6–8), n (%)*	13 (1.3)	6 (3.3)	0.149

***Odds ratio for FH detection (95% CI)***	Reference	3.59 (1.84–6.99)	


FH, familial hypercholesterolemia; DLCN, Dutch Lipid Clinic Network; CI, confidence interval. Data are presented as n (%). P-values were calculated using the chi-square test.

### Community-based screening outcomes

**Figure 2:**
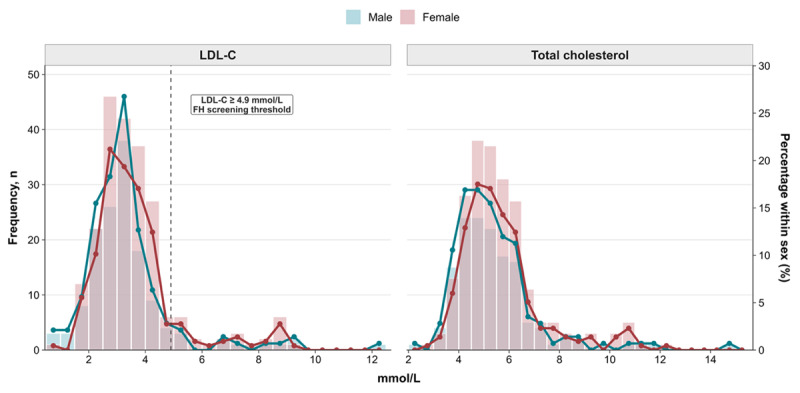
Distributions of cholesterol levels in community-based screening linked to an index case. FH, familial hypercholesterolemia; LDL-C, low-density lipoprotein cholesterol.

The screened population had a median age of 47 years, with females accounting for 60.4% of participants. The distributions of total cholesterol and LDL-C levels are shown in Figure 2. The mean total cholesterol and LDL-C levels were 5.49 ± 1.65 mmol/L (95% CI 5.32–5.66) and 3.47 ± 1.56 mmol/L (95% CI 3.30–3.63), respectively. Mean total cholesterol and LDL-C levels did not differ significantly between males and females (5.32 ± 1.7 vs. 5.61 ± 1.61 mmol/L and 3.29 ± 1.6 vs. 3.6 ± 1.5 mmol/L, respectively; Student’s t-test, p > 0.05).

Overall, 10.6% of participants had LDL-C levels ≥ 4.9 mmol/L, with comparable proportions in males and females (9.2% vs. 11.5%, chi-square test, p > 0.05).

The community-based program successfully extended the screening to younger age groups. Among 84 screened young adults, six (7.1%) had LDL-C levels ≥ 4.9 mmol/L. In addition, screening of 56 children identified five cases (8.9%) with LDL-C levels ≥ 4.9 mmol/L.

## Discussion

The results of this study demonstrated the feasibility and real-world applicability of a stepwise targeted FH screening strategy in a resource-limited setting, initiated in patients with premature CAD and subsequently extended to a geographically defined community linked to an index case. This approach was associated with an increased detection yield in selected high-risk populations and enabled the identification of individuals with markedly elevated LDL-C levels across their life course. However, considering the observational design and the selected nature of the study populations, these findings should be interpreted as indicative of feasibility and implementation potential. Together, these findings support the potential for structured and scalable FH detection pathways in LMICs. This study therefore provides implementation-relevant evidence addressing a key translational gap between guideline recommendations and real-world practice.

### Targeted hospital-based screening as an entry point for FH detection

The prevalence of phenotypic FH among patients with premature CAD ranged from 2.5% in retrospective screening to 8.3% in prospective screening, representing an approximately 10- to 30-fold enrichment compared with general population estimates (1 in 250–311) (10). This enrichment confirms that premature CAD is a highly efficient clinical entry point for FH detection and supports risk-based screening strategies in settings where universal screening is not feasible. Our findings are consistent with those of previous studies reporting elevated FH prevalence in premature CAD cohorts, including a Vietnamese study that reported potential FH in 14.7% of patients with premature myocardial infarction (11).

Prospective hospital-based screening achieved a higher detection yield of phenotypic FH compared with retrospective screening (8.3% vs. 2.5%), which is partly attributable to more systematic and standardized phenotyping, including more complete ascertainment and documentation of key DLCN components such as family history and physical signs. This interpretation is supported by the observed differences in the availability of these components between cohorts (Supplementary Table S1).

Although both cohorts were drawn from the same underlying population and applied the same diagnostic criteria, differences in data completeness and ascertainment likely contributed to the observed discrepancy in detection yield. The prospective design enabled structured data collection and active case finding, whereas the retrospective cohort was inherently limited by underdocumentation of key DLCN components, particularly family history and physical signs, in routine health records, which is a common challenge in secondary data analysis. Prospective screening also facilitates structured patient interviews and cascade screening, whereas retrospective analyses are more susceptible to incomplete documentation and potential selection bias (12).

Because this was an observational comparison between non-concurrent cohorts, these findings should be interpreted as reflecting differences in case ascertainment and data completeness between study components. Accordingly, the higher detection yield observed in the prospective cohort is more likely attributable to improved ascertainment rather than a true difference in underlying disease prevalence. These findings should be interpreted based on differential data completeness between study components, which may have contributed to FH underestimation in the retrospective cohort. Nevertheless, the retrospective detection rate remains clinically meaningful and demonstrates the feasibility of leveraging routine hospital databases for large-scale identification of FH cases in LMICs.

These findings align with the World Health Organization (WHO) Global Action Plan for the Prevention and Control of Noncommunicable Diseases (NCDs) 2013–2030, which emphasizes the early identification of high-risk individuals and the strengthening of health information systems for cardiovascular disease prevention. Embedding automated or clinician-triggered FH screening algorithms in electronic health records could further enhance early detection and facilitate systematic cascade screening within national NCD programs (13).

### Community-based screening linked to index cases: detection beyond clinical settings

Extending screening to community members linked to an index case in the present study enabled the identification of individuals with markedly elevated LDL-C levels, including young adults and children. This underscores the importance of cascade screening that leverages the inherited nature of FH to systematically identify affected relatives and enable early preventive interventions. Early detection in younger individuals is particularly important, as timely lipid-lowering therapy substantially reduces the lifetime cardiovascular risk.

Cascade screening using cholesterol testing, genetic testing, or both has been recommended by national and international organizations and classified by the US Centers for Disease Control and Prevention as a Tier 1 genomic application, with grade A evidence for clinical and public health integration (14). In Vietnam, the VINAFH program demonstrated the feasibility and effectiveness of cascade screening in a real-world setting (6). Aggregation of FH phenotypes within defined communities demonstrates the potential for geographically targeted screening linked to index cases as an intermediate and resource-efficient strategy between cascade and universal screening, particularly in LMIC contexts where universal screening remains challenging (15).

The rationale for geographically targeted screening linked to an index case is grounded in the inherited nature of FH and clustering of biologically related individuals within the same household or community in LMIC settings. This approach leverages the high likelihood that first- and second-degree relatives reside in close proximity, thereby enriching the screened population for individuals at increased genetic risk. Therefore, geographically targeted screening represents a pragmatic extension of cascade screening, enabling more efficient case finding beyond the immediate family while remaining more resource-efficient.

Community-based screening was able to reach younger populations in the present study, with elevated LDL-C levels detected in young adults and children. These findings highlight the potential of geographically targeted cascade screening to identify undiagnosed FH across generations. The participation rate (59.1%) observed in the community screening component indicates moderate uptake for a population-based initiative in a resource-limited setting and should be interpreted cautiously, reflecting the effectiveness of community engagement strategies and logistical and contextual barriers inherent to large-scale screening programs. Non-participation may have been influenced by several factors, including limited FH awareness, perceived lack of symptoms, competing work or family commitments, accessibility constraints related to the timing and location of screening activities, and sociocultural and health-seeking factors. These considerations underscore the need for tailored community engagement strategies, flexible delivery models, and integration with primary care services to improve participation and support the scalability of community-based FH screening programs. Moreover, the observed prevalence in this setting may be influenced by participation bias and familial clustering and should not be extrapolated to the general population.

However, the community screening was conducted in a geographically defined population linked to a single index case, limiting generalizability and possibly enriching the observed prevalence owing to clustering of related individuals. LDL-C thresholds alone may not fully distinguish monogenic FH from polygenic or secondary hypercholesterolemia, particularly in adults. Genetic testing of index cases can improve cascade screening accuracy and inform precision prevention strategies, although the absence of a detectable genetic mutation does not exclude FH. The limited use of genetic testing constrains the ability to distinguish monogenic FH from other causes of hypercholesterolemia and can lead to misclassification in phenotypic assessment. Improving participation is critical for scaling community-based FH screening within national NCD programs.

### Policy implications and public health impact

From a public health perspective, our findings support a stepwise FH detection framework aligned with the WHO Package of Essential Noncommunicable (PEN) Disease for Primary Health Care. Targeted screening of patients with premature CAD, followed by cascade and geographically targeted community screening, may be a pragmatic and scalable model for LMICs. Economic evaluations have shown that cascade screening is more cost-effective than not screening at all, and modeling studies suggest that targeted screening strategies can lead to substantial health gains at a relatively low cost (16,17). Integrating FH detection into national NCD programs, primary care pathways, and family-based prevention frameworks could enable earlier diagnosis, improve treatment uptake, and reduce premature cardiovascular morbidity and mortality. In settings with constrained resources, a stepwise targeted approach may provide an actionable pathway to bridge the current FH diagnostic gap while building capacity for broader population-based strategies.

### Strengths and limitations

The strengths of this study include its stepwise design linking hospital- and community-based screening, the use of standardized phenotypic criteria, and the implementation of a multilevel implementation framework involving national, provincial, and community stakeholders.

Several limitations should be considered. First, the hybrid retrospective-prospective design introduced non-comparability between cohorts due to incomplete documentation and varying data quality in legacy medical records. Second, genetic testing was performed only in a limited subset of participants, restricting molecular confirmation; thus, reliance on phenotypic criteria alone may have led to potential misclassification between monogenic FH and other etiologies, such as polygenic hypercholesterolemia. Third, the definition of chronic CAD based on ≥ 50% stenosis may have excluded some patients with non-obstructive CAD. In addition, community-based screening was conducted in a geographically restricted population linked to a single index case, limiting generalizability. Moreover, non-participation in the community screening component may also have introduced selection bias, affecting the representativeness of the screened population. Future studies should evaluate long-term clinical outcomes, cost-effectiveness, and implementation metrics to inform broader scale-up.

## Conclusions

Targeted screening of patients with premature CAD, followed by cascade and geographically targeted community screening, represents a feasible and potentially scalable strategy for FH detection in LMICs. Premature CAD is a highly enriched clinical phenotype for FH, and prospective systematic screening may improve case detection. Leveraging hospital records for retrospective case identification, combined with prospective phenotypic assessment using the DLCN criteria and community-based screening linked to index cases, may provide a pragmatic and context-specific pathway for integrating FH detection into national NCD programs and warrants further evaluation in larger and more representative populations.

**Supplementary Table S1:** Availability and distribution of selected DLCN components in retrospective and prospective hospital-based cohorts.


CHARACTERISTICS	RETROSPECTIVE SCREENING (n = 972)	PROSPECTIVE SCREENING (n = 180)

***Family history***		

*First-degree relative with premature CAD and/or vascular disease*	1 (0.1)	9 (5)

*First-degree relative with known LDL-C > 95th percentile for age and sex*	1 (0.1)	6 (3.3)

*First-degree relative with tendon xanthoma and/or arcus cornealis*	N/A	0

*Children < 18 years with known LDL-C > 95th percentile for age and sex*	N/A	1 (0.6)

***Clinical history***		

*Patient with premature cerebral or peripheral*	1 (0.1)	3 (1.7)

***Physical Examination***		

*Tendon xanthomata*	N/A	7 (3.9)

*Arcus cornealis at age < 45 years*	N/A	10 (5.6)


CAD, coronary artery disease; DLCN, Dutch Lipid Clinic Network; LDL-C, low density lipoprotein-cholesterol. Data are presented as n (%). “N/A” indicates that the variable was not systematically recorded in the retrospective cohort. Due to the retrospective design, documentation of several DLCN components—particularly family history and physical examination findings—was limited, which may have led to under-ascertainment in the retrospective cohort.

## Data Availability

All data generated or analyzed during this study are included in the published article. Further details are available from the corresponding authors upon reasonable request.
